# Testing the Effects of Prey Type on the Life History and Population-Level Parameters of *Chrysoperla externa* (Neuroptera: Chrysopidae)

**DOI:** 10.3390/insects15050330

**Published:** 2024-05-03

**Authors:** Agda Braghini, Vinícius de Oliveira Lima, Bruno Gomes Dami, Jonas Mendes Rodrigues Souza, Enes Pereira Barbosa, Gustavo Pincerato Figueiredo, Wesley Bordinhon da Silva Paula, Cesar Rodriguez-Saona, Alessandra Marieli Vacari

**Affiliations:** 1 Laboratory of Entomology, University of Franca (UNIFRAN), Avenida Dr Armando Sales de Oliveira, 201, Parque Universitário, Franca 14404-600, SP, Brazil; agdabraguine@hotmail.com (A.B.); volima_2013@hotmail.com (V.d.O.L.); bruno_g.dami@hotmail.com (B.G.D.); jonasmendesrs97@hotmail.com (J.M.R.S.); enes.organicos@gmail.com (E.P.B.); gustavopincerato@bol.com.br (G.P.F.); wesley-silva1994@hotmail.com (W.B.d.S.P.); 2 Yara Brazil, Rua Diogo Moreira, 184, São Paulo 05423-010, SP, Brazil; 3 Abrafol, Rua Antônio Ribas, 391, Distrito Industrial, Brodowski 14340-000, SP, Brazil; 4 EMATER, Claraval 37997-000, MG, Brazil; 5 GPF Agricultural Research, Cristais Paulista 14460-000, SP, Brazil; 6 P.E. Marucci Center, Rutgers University, 125A Lake Oswego Rd., Chatsworth, NJ 08019, USA; crodriguez@njaes.rutgers.edu

**Keywords:** green lacewing, life table parameters, predator–prey interactions, augmentative biological control

## Abstract

**Simple Summary:**

Green lacewings, particularly *Chrysoperla externa*, play a pivotal role in pest control within agriculture. In our study, we examined the performance of *C. externa* when fed on the following three distinct prey types: the pupae of the coffee leaf miner, the eggs of the sugarcane borer, and the eggs of the Mediterranean flour moth. Our aim was to ascertain how prey type influences lacewing development and population dynamics. We conducted experiments in a controlled environment, assessing variables such as survival rates, developmental duration, adult lifespan, and reproductive capability. The results demonstrated that *C. externa* exhibits robust performance when consuming natural prey items, such as coffee leaf miner pupae and sugarcane borer eggs. Interestingly, the predator exhibited even greater success when fed Mediterranean meal moth eggs, despite this not being its natural prey. This finding underscores the adaptability of lacewings to a broad range of prey items that are still conducive to their growth and reproduction. Understanding the dietary preferences and responses of lacewings to different prey types informs the optimization of their utilization in agricultural pest management programs. It offers valuable insights into the development and reproductive behavior of predators consuming natural prey, thus enhancing the efficacy of pest control strategies.

**Abstract:**

Green lacewings are valuable predators, utilized in augmentative biological control against various agricultural pests. However, further studies are required to comprehend the performance of these predators when consuming natural prey. We investigated the capacity of *Chrysoperla externa* (Hagen) to utilize the following three distinct prey types: the pupae of the coffee leaf miner *Leucoptera coffeella* (Guérin-Mèneville & Perrottet), the eggs of the sugarcane borer *Diatraea saccharalis* (F.), and the eggs of the Mediterranean flour moth *Ephestia kuehniella* (Zeller). The first two of these species are naturally occurring prey found in field crops, while the last serves as a factitious prey species for the mass rearing of natural enemies. We hypothesized that the type of prey would differentially affect the life history and population-level parameters of *C. externa*. Laboratory experiments were conducted to compare the pre-imaginal survival and developmental times, adult longevity and reproduction, and population growth of *C. externa* when larvae were provided with each of the three prey items. Results indicated that *C. externa* utilized the two natural prey items, *L. coffeella* pupae and *D. saccharalis* eggs, for its development, reproduction, and population growth. However, larvae developed significantly faster and females exhibited higher reproductive parameters, including fecundity and daily oviposition, when consuming the factitious prey, *E. kuehniella* eggs. This resulted in a higher intrinsic rate of population increase, as well as shorter times for the population to double in size. Understanding the population dynamics of *C. externa* when consuming different prey items is crucial for optimizing their utilization in augmentative biological control programs.

## 1. Introduction

Green lacewings are among the most important and frequently utilized natural enemies in the biological control of agricultural pests [1]. They possess numerous attributes that make them excellent biological control agents and prime candidates for augmentative releases. These include their propensity to prey on a wide range of soft-bodied insects, such as aphids, whiteflies, thrips, and lepidopterans, which are commonly targeted pests in biological control programs [2,3]. Another advantageous trait is that, while adults primarily feed on nectar, pollen, and honeydew [4], the larvae exhibit high mobility and voracious predatory behavior, with their predatory capacity increasing as they mature [5,6]. Furthermore, green lacewings are easily reared in laboratory settings [7] and are currently being deployed as control agents for numerous agricultural crops worldwide. This includes widespread usage across several South American countries, such as Brazil, Argentina, Peru, and Colombia [8,9,10,11].

Notably, *Chrysoperla externa* (Hagen) (Neuroptera: Chrysopidae) exhibits high adaptability to various climates, enabling it to enjoy a wide geographical distribution [12]. As one of the most commonly found lacewing species in the Americas, it ranges from the southern United States to Argentina [12,13]. In Brazil, five companies currently hold registrations with the Ministry of Agriculture, Livestock, and Supply for lacewing releases, with more expected to follow suit. This trend is expected to significantly expand the area treated with these predators, which increased from 60,000 ha/year in 2022 to approximately 150,000 ha/year in 2023 [14]. Despite the ease of rearing this predator in the laboratory, challenges persist in determining whether predators released in the field can survive and reproduce, thereby increasing their population solely through the consumption of natural prey.

To gain a comprehensive understanding of the performance of *C. externa* when consuming natural prey, research on its life history is crucial. In fact, despite the potential significance of this predator as a biological control agent [15,16], studies elucidating its life history and population-level parameters have been scarce. Addressing this gap, we conducted laboratory studies aiming to unravel the pre-imaginal development, survival, life table parameters, fecundity, and adult longevity of *C. externa* when consuming the following three prey items: the pupae of the coffee leaf miner *Leucoptera coffeella* (Guérin-Mèneville & Perrottet) (Lepidoptera: Lyonetiidae), the eggs of the sugarcane borer *Diatraea saccharalis* (F.) (Lepidoptera: Crambidae), and the eggs of the Mediterranean flour moth *Ephestia kuehniella* (Zeller) (Lepidoptera: Pyralidae). The eggs of *E. kuehniella* are commonly utilized as factitious prey items in the mass rearing programs of natural enemies [17], which is why we used this prey for comparison purposes. *Leucoptera coffeella* is considered one of the most economically significant pest species of coffee in Brazil [18]; the exposed nature of the pupal stage renders it readily accessible for *C. externa* consumption, unlike eggs and larvae within the mines [15]. The eggs of *D. saccharalis*, a key sugarcane pest in South America, can also serve as natural prey for green lacewing larvae [19].

In this study, we examined the life history and population-level parameters of *C. externa* when provided with the eggs of *D. saccharalis* and *E. kuehniella*, as well as the pupae of *L. coffeella* (Figure S1). We selected the eggs of *D. saccharalis* and *E. kuehniella* as prey items due to their common use in rearing programs. The pupae of *L. coffeella* were chosen as the most suitable stage for *C. externa* consumption because of their exposed nature, unlike the eggs and larvae, which are hidden within the mines. We hypothesized that prey type (factitious versus natural) has differential effects on the pre-imaginal development, survival, reproduction, and adult longevity of the predator *C. externa*. This research will contribute to our comprehension of the population dynamics of *C. externa* when consuming different prey items, thereby enhancing the effectiveness of integrating augmentative biological control involving *C. externa* into broader Integrated Pest Management (IPM) programs.

## 2. Materials and Methods

### 2.1. Rearing of Chrysoperla externa

Green lacewing adults, *C. externa*, were initially collected from organic coffee plantations in the Franca region, São Paulo, Brazil (latitude 20°32′19″ S, longitude 47°24′03″ W). They were then transported to the laboratory and subsequently used for experiments. All individuals were kept in the laboratory under controlled conditions, as follows: 25 ± 1 °C, a 12L:12D photoperiod, and 70 ± 10% relative humidity. The colony was maintained following methods adapted from Finney [20] and Freitas [21]. The adults were housed in rearing cages (N = 20), constructed from cylindrical transparent plastic containers measuring 15 cm in diameter and 20 cm in height. These dimensions accommodated letter-size bond paper, which served as a substrate for oviposition. Two openings were made in the upper part of the container: one with a diameter of 4 cm, covered with voile tissue for aeration, and another for placing a plastic container (28 mm in diameter and 15 mm in height) containing the diet for the adults. The diet, comprising honey and brewer’s yeast at a 1:1 ratio, was provided using a sponge slightly larger than the lid to ensure attachment. Additionally, a piece (2 cm × 2 cm) of sponge soaked in deionized water was placed at the bottom of each cage.

Colony maintenance was conducted every 2 days, involving the replacement of diet and water, as well as the removal of deceased adults. The sex ratio was maintained at 1:4 male to female, resulting in 3 males and 12 females per cage. During colony maintenance, the paper sheet used for oviposition was replaced with a new one, and subsequently cut into pieces containing defined amounts of eggs. Initially, predators were collected from the field and subsequently cultured in the laboratory using *E. kuehniella* eggs for one generation to obtain the required number of individuals for the experiments. Following one generation of predator rearing in the laboratory, *C. externa* individuals were utilized for the experiments. After egg collection, each egg was placed in a transparent plastic container measuring 6 cm in diameter and 3 cm in height. Eggs typically hatched within 3–5 days. Subsequently, eggs of *D. saccharalis* and *E. kuehniella*, along with pupae of *L. coffeella* (less than 24 h old), were provided as prey for the lacewing larvae. Prey items were replenished every 2 days. Individuals were maintained in the containers throughout both the larval and pupal stages. Upon emergence, adults of the same age were transferred to cages as described earlier. Approximately 5 days after emergence, new cages were assembled with the previously mentioned male to female densities. Specimens from the colony underwent species confirmation by a taxonomist (Dr. Francisco José Sosa Duque, Universidade Federal Rural da Amazônia, Capitão Poço, PA, Brazil).

### 2.2. Rearing and Sources of Prey

*Leucoptera coffeella* individuals were obtained from a laboratory colony, which was initiated from coffee leaves containing live larvae and pupae of *L. coffeella*, collected from organic coffee plantations in the Franca region, São Paulo, Brazil (20°27′27″ S, 47°35′24″ W). The larvae and pupae were placed in transparent plastic containers (12 cm in diameter × 15 cm in height). Upon emergence, adults were separated by sex according to Motta et al. [22], and couples were formed. Coffee plants containing 4 to 5 pairs of leaves were used to house the couples. Each leaf of the coffee plant was enclosed in a voile fabric bag (10 cm in length × 6 cm in width), each accommodating two males and two females, and the bag was secured with a string on the branch. The coffee plants hosting *L. coffeella* adults were maintained in the laboratory under controlled conditions (25 ± 1 °C, a 12L:12D photoperiod, and 70 ± 10% relative humidity). Leaves were inspected daily for *L. coffeella* larvae and pupae, which were then removed and individually placed in plastic tubes (2.5 cm in diameter × 6 cm in height) until adult emergence. After emergence, adults were again separated by sex, and couples were placed with a clean, undamaged coffee leaf to serve as an oviposition substrate for the females. The adult insects were provided with a 10% honey solution, administered through a moistened sponge cube (1 cm^2^) placed inside the voile bag.

*Ephestia kuehniella* larvae were cultured in the laboratory and fed a diet comprising whole wheat flour (97%) and brewer’s yeast (3%). The whole wheat flour underwent sterilization at 150 °C for two hours, with yeast added after cooling. This diet was then transferred to plastic containers (47 cm × 29.5 cm × 10.5 cm), with 1 kg of diet evenly distributed in each container. Four shallow grooves were created in the diet, aligned parallel to each other along the length of the container, where the eggs were evenly dispersed. The eggs were used in a ratio of 0.15 g per kilogram of diet. The rearing container was covered with a lid featuring a rectangular opening (6 cm × 8 cm) covered with voile fabric, following the methodology of Parra et al. [23] with modifications. Adults were collected daily using a modified vacuum cleaner (Powerspeed Plus STK14 1300W, Electrolux, Curitiba, PR, Brazil), equipped with a capture chamber made from a polyethylene terephthalate (PET) bottle and polyvinyl chloride (PVC) pipe. Adult cages were constructed using cylindrical acrylic containers (20 cm in diameter × 35 cm in height), with 60 females and 40 males housed in each container. Screen houses, folded in a “Z” shape, were placed inside these cages as a substrate for oviposition. The cage was closed with another screen house, secured with elastic glue. Egg collection was carried out daily by inverting the cage over a white plastic container and shaking it so that the eggs attached to the substrate fell into the container. Subsequently, the eggs were sifted to remove impurities, with a portion allocated for predator rearing and the remainder for maintaining the *E. kuehniella* colony. Excess eggs were stored in the refrigerator at 6 °C for later use. The colony was sustained in a climate-controlled room, with a temperature of 28 ± 1 °C, a photoperiod of 12L:12D, and a relative humidity of 70 ± 10%. *D. saccharalis* eggs were sourced from the Entomology Laboratory of São Martinho Sugar Mill, Pradópolis, SP, Brazil.

### 2.3. Pre-Imaginal Development and Survival of Chrysoperla externa

To initiate the experiments on the pre-imaginal development and survival of *C. externa* when consuming different prey items, predator eggs were gathered from the laboratory colony and individually stored in Eppendorf tubes. All eggs were collected on the same day and were less than 12 h old. Every 2 h, the eggs were inspected, and newly emerged larvae were individually transferred, using a fine brush, to transparent plastic containers (6.0 cm in diameter × 3.0 cm in height). To ensure adequate ventilation within the container, a 1.5 cm × 1.5 cm opening was created on the side and covered with voile fabric. Approximately 100 *D. saccharalis* eggs were provided daily for each *C. externa* larva during its larval development. For *L. coffeella*, 10 pupae were introduced daily to each container. In consideration of alternative prey species, a constant surplus of frozen *E. kuehniella* eggs (0.5 g) was offered daily to each *C. externa* larva.

Every 24 h, the larval developmental stage and survival were documented. The experiment was conducted in a climate-controlled room maintained at a temperature of 25 ± 1 °C, with a 12L:12D photoperiod, and a relative humidity of 70 ± 10%. For each treatment (prey species tested), 80 newly emerged *C. externa* larvae were utilized, with only those that completed their development being included in the data analysis. The experimental design employed was completely randomized, with each *C. externa* larva considered a replicate (N = 80).

### 2.4. Longevity and Fecundity of Chrysoperla externa

Females and males of *C. externa*, which had been fed either *D. saccharalis* eggs, *E. kuehniella* eggs, or *L. coffeella* pupae, were paired as couples on the day of emergence and housed in transparent cylindrical plastic cages (15 cm in diameter × 20 cm in height). These cages provided continuous access to water and a liquid diet consisting of honey and brewer’s yeast at a 1:1 ratio. All surviving adults from the previous experiment were utilized to form each couple. Female survival and the number of eggs laid by each female were recorded daily. In cases where males died, they were replaced by new ones to ensure continuous mating.

The egg hatching rate was estimated following a method similar to that described by Pappas et al. [24]. Over the first 30 days of the oviposition period, 100 eggs were randomly sampled from each female and individually transferred to Eppendorf tubes. These eggs were maintained under the same temperature and abiotic conditions as the parental females, with daily evaluations conducted to record the number of newly emerged larvae. The egg hatching rate was then estimated by calculating the percentage of emerged larvae in each treatment based on the total number of eggs laid. The experimental design was completely randomized, with each *C. externa* couple considered a replicate.

### 2.5. Data Analyses

All analyses were conducted using SAS software version 9.4 [25]. Data on the pre-imaginal development, longevity, and fecundity of *C. externa* were checked for normality using the Shapiro–Wilk test [26] and homoscedasticity using the Bartlett test [27], as required for analysis of variance (ANOVA) (PROC UNIVARIATE). Since the data on larval, pupal, and pre-imaginal period duration did not meet the assumptions of normality and homoscedasticity, they were analyzed using the Kruskal–Wallis H test (PROC NPAR1WAY) [28], followed by the Dwass–Steel–Critchlow–Fligner (DSCF) test for multiple comparisons (α = 0.05). ANOVA was used to compare the effects of prey species on the female longevity, male longevity, fecundity (eggs/female), pre-oviposition, and oviposition periods of *C. externa* adults. To meet the assumptions of ANOVA, the longevity of males and fecundity of females were transformed using the square root of x + 0.5. When significant differences were observed, means were compared using the Student–Newman–Keuls test (PROC ANOVA; α = 0.05). The pre-imaginal survival and egg hatching rate of *C. externa* fed different prey species were analyzed using logistic regression (PROC GENMOD; α = 0.05). The percentage survival (egg until adult death) of *C. externa* fed different prey species was analyzed using Cox regression (PROC PHREG; α = 0.05) [29].

Life table parameters and fertility were estimated by combining data from the pre-imaginal developmental experiment, the survival of pre-imaginal and adult stages, and reproduction, following the methodology outlined by Birch [30] and Southwood and Henderson [31]. These parameters include the following: x = the mean age of parent females, lx = survival until age x, mx = specific fertility, and lx.mx = total number of females born at age x. Growth parameters obtained from the life table were calculated as follows: *R*_0_ = net reproductive rate, *T* = mean generation time, r_m_ = intrinsic rate of population growth, and λ = finite rate of population growth. Additionally, *D*t, representing the time required for the population to double in number, was determined according to Krebs [32].

The Jackknife method was employed to estimate the means and standard errors. Life table parameters were estimated in accordance with Maia et al. [33], utilizing PROC LIFETEST. Mean values of the life table parameters were compared using the Student’s *t*-test (*p* = 0.05).

## 3. Results

### 3.1. Pre-Imaginal Development and Survival of Chrysoperla externa

The duration of the egg stage in *C. externa* ranged from 4.7 to 5.8 days. The larval period was significantly influenced by the prey item consumed. *Chrysoperla externa* exhibited the shortest larval period when consuming *E. kuehniella* eggs (Table 1), with this parameter being 9.6 days shorter, when compared to the consumption of *L. coffeella* pupae, than that of *D. saccharalis* eggs (χ^2^ = 43.71; DF = 2; *p* < 0.001). Conversely, the pupal stage in *C. externa* was shortest when consuming *L. coffeella* pupae, showing a pupal period that was 2.3 and 3.8 days shorter compared to consumption of *E. kuehniella* and *D. saccharalis* eggs, respectively (χ^2^ = 21.64; DF = 2; *p* < 0.001). Consequently, the entire pre-imaginal period of *C. externa* was shortest when consuming *E. kuehniella* eggs, but it was also 10.6 days shorter when consuming *L. coffeella* pupae compared to *D. saccharalis* eggs (χ^2^ = 43.52; DF = 2; *p* < 0.001) (Table 1).

**Figure 1:**
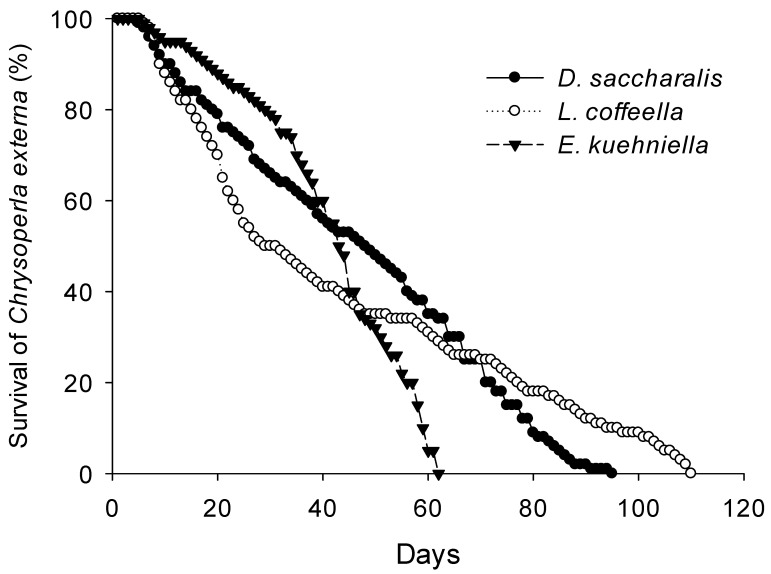
Percentage survival (egg until adult death) of *Chrysoperla externa* preying on *Diatraea saccharalis* eggs, *Leucoptera coffeella* pupae, and *Ephestia kuehniella* eggs (N = 80 green lacewings per prey item) (Cox regression, *p* < 0.05).

The survival of *C. externa* throughout the entire pre-imaginal period, spanning from egg to adult, was not significantly influenced by the consumption of different prey items (χ^2^ = 4.69; DF = 2; *p* = 0.0959) (Table 1). However, larvae that consumed *E. kuehniella* eggs had shorter lifespans, from egg until adult death, than those that consumed *L. coffeella* pupae or *D. saccharalis* eggs (Figure 1).

### 3.2. Longevity and Fecundity of Chrysoperla externa

The longevity of *C. externa* females was notably shortened when they consumed *E. kuehniella* eggs as larvae (F = 14.62; DF = 2.28; *p* < 0.001) (Table 2). Similarly, the longevity of *C. externa* males was influenced by prey item, with males from larvae that consumed *L. coffeella* pupae exhibiting a lifespan 44.2 days longer than those that consumed *D. saccharalis* eggs (F = 11.45; DF = 2.31; *p* < 0.001) (Table 2). However, the fecundity of *C. externa* females was not significantly influenced by the consumption of different prey items during the larval stage (F = 2.91; DF = 2.28; *p* = 0.081) (Table 2). In contrast, the egg hatching rate was 41% higher when *C. externa* consumed *L. coffeella* pupae compared to *E. kuehniella* eggs (χ^2^ = 21.11; DF = 2; *p* < 0.001) (Table 2).

The pre-oviposition period of *C. externa* females was unaffected by prey item (F = 0.58; DF = 2.28; *p* = 0.573). However, when predators consumed *L. coffeella* pupae, the oviposition period was 26 days and 33 days longer, compared to when consuming *D. saccharalis* eggs and *E. kuehniella* eggs, respectively (F = 4.17; DF = 2.28; *p* = 0.032) (Table 3).

### 3.3. Demographic Parameters

The consumption of different prey items by *C. externa* significantly influenced their life table parameters (Table 4). High values of *R*_0_, *r*_m_, and λ were recorded when larvae were fed with *E. kuehniella* eggs, compared to *D. saccharalis* eggs and *L. coffeella* pupae. However, concerning *R*_0_, both *D. saccharalis* eggs and *L. coffeella* pupae emerged as potential prey items, fostering a substantial increase in *C. externa* population size. The lower values of population growth (*r*_m_ and λ) found when *C. externa* consumed *D. saccharalis* eggs and *L. coffeella* pupae are likely attributed to higher larval mortality. The mean generation time (*T*) was 18.8 days longer when *C. externa* consumed *D. saccharalis* eggs than when they consumed *E. kuehniella* eggs. Despite these differences, all prey items studied allowed the *C. externa* population to grow (i.e., *r*_m_ > 0), but with a considerably longer doubling time (*Dt*) when *D. saccharalis* eggs were provided as food, compared with *E. kuehniella* eggs.

**Figure 2:**
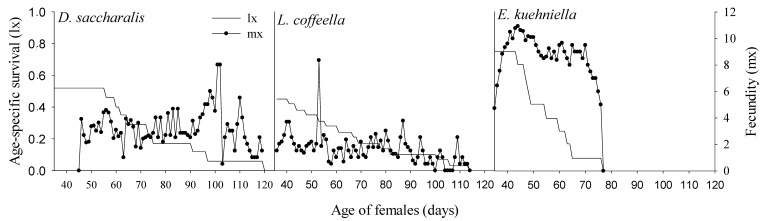
Age-specific survival (lx, number of females alive at age x) and age-specific fecundity (mx, number of female offspring produced by females at age x) of *Chrysoperla externa* preying on *Diatraea saccharalis* eggs, *Leucoptera coffeella* pupae, and *Ephestia kuehniella* eggs.

The female progeny production by *C. externa* was 36% higher when the predator consumed *E. kuehniella* eggs, compared to when they fed on *D. saccharalis* eggs or *L. coffeella* pupae (Figure 2). While age-specific fecundity was highest when *C. externa* larvae consumed *E. kuehniella* eggs, females exhibited shorter lifespans compared to when they fed on *D. saccharalis* eggs and *L. coffeella* pupae. *Chrysoperla externa* lived a maximum of 77, 114, and 120 days when they consumed *E. kuehniella* eggs, *L. coffeella* pupae, and *D. saccharalis* eggs, respectively (Figure 2). Fifty percent mortality occurred at 48, 55, and 35 days when *C. externa* larvae consumed *E. kuehniella* eggs, *D. saccharalis* eggs, and *L. coffeella* pupae, respectively (Figure 2).

## 4. Discussion

The green lacewing, *C. externa*, successfully utilized both natural prey items provided—*L. coffeella* pupae and *D. saccharalis* eggs—for development and reproduction. However, it developed significantly faster and exhibited higher reproductive parameters, such as fecundity and daily oviposition, when larvae consumed the factitious prey, *E. kuehniella* eggs. Despite the shorter pupal and longer oviposition times observed when *C. externa* consumed *L. coffeella* pupae, there was no increase in the numbers of eggs produced per female. Instead, larval consumption of *E. kuehniella* eggs led to significantly higher *C. externa* life table parameters. For example, the time needed for *C. externa* to double in population size was shorter when larvae consumed *E. kuehniella* eggs, compared to when they fed on *D. saccharalis* eggs or *L. coffeella* pupae. Under our experimental conditions, we draw the following two main conclusions: (a) *E. kuehniella* eggs are the most favorable prey item among those tested for *C. externa* mass rearing, and (b) *C. externa* larvae can utilize *D. saccharalis* eggs and *L. coffeella* pupae as viable natural prey items.

While previous studies have demonstrated that *C. externa* can feed and reproduce on *D. saccharalis* and *E. kuehniella* eggs [34,35,36], to our knowledge, this is the first investigation revealing the developmental and reproductive success of *C. externa* on *L. coffeella* pupae. It is noteworthy that previous studies did not report life table parameters for *C. externa*, hindering direct comparisons of predator performance among different prey items. Our study fills this gap, allowing for a more comprehensive assessment. The net reproductive rates (R0) of *C. externa*, when provided with *E. kuehniella* eggs (149.0 females/female), were in proximity to values reported by Palomares-Pérez et al. [37], when predators consumed sugarcane aphids, *Melanaphis sacchari* (Zehntner) (113.2 females/female), but approximately three times higher than when they consumed *L. coffeella* (46.2 females/female) and *D. saccharalis* (51.8 females/female). Even though *C. externa* performance was comparatively lower when consuming the two natural prey items—*L. coffeella* pupae and *D. saccharalis* eggs—than when fed on the factitious prey item, *E. kuehniella* eggs, an increase in the predator’s population size was still observed. This indicates the suitability of *L. coffeella* pupae and *D. saccharalis* eggs as prey items. Despite these promising findings, further studies assessing the efficacy of *C. externa* as a biological control agent for *L. coffeella* and *D. saccharalis* under field conditions are imperative.

In Brazil, natural populations of *C. externa* are commonly found in coffee and sugarcane fields, where *L. coffeella* and *D. saccharalis*, respectively, occur; therefore, this predator species is acclimated to surviving under the hot and humid conditions typical of these crops [38,39]. Furthermore, *C. externa* from commercial sources, i.e., individuals reared under laboratory conditions, exhibit robust performance when deployed in augmentative biological control programs within these hot and humid environments [9]. The natural occurrence of *C. externa* in these agricultural settings, and their successful performance under both natural and laboratory conditions, underscores their inherent acclimation to the specific climatic nuances associated with coffee and sugarcane cultivation. This establishes a strong foundation for considering *C. externa* as an effective and readily adaptable biological control solution against pests in these crops.

The larvae of *C. externa* exhibit a versatile feeding behavior as generalist predators, capable of preying on a diverse array of insects, including aphids, scale insects, leafhoppers, whiteflies, thrips, mites, and the eggs and larvae of lepidopterans [40]. For other species of lacewings, studies have shown that the type of prey consumed during the larval stage significantly influences various aspects of the predator’s life cycle [24,41,42,43,44]. In the present study, we demonstrated that the type of diet provided during the larval stage had strong effects not only on *C. externa* pre-imaginal development and survival, but also on adult longevity and fecundity. Notably, the prey item that facilitated rapid pre-imaginal development and high survival rates for *C. externa* also correlated with superior reproductive performance.

In conclusion, *C. externa* exhibited successful development and reproduction when provided with all three studied prey items—*D. saccharalis* eggs, *L. coffeella* pupae, and *E. kuehniella* eggs. However, among these prey items, *E. kuehniella* eggs were the most favorable for the development and reproduction of *C. externa*, and could thus be used for mass rearing and augmentative releases of this predator. In our experiments, the total prey consumption by *C. externa* was not quantified. To refine our understanding, future studies should focus on determining the quantity of prey consumed by larvae when provided with different prey items. Under field conditions, *C. externa* larvae are likely to encounter a variety of prey. Therefore, further investigations are essential to elucidate whether diets incorporating different prey items influence the predator’s preference, performance, and life table parameters. This study sheds light on the potential of this predator as a biological control agent for *D. saccharalis* and *L. coffeella*. Such insights pave the way for a broader adoption of augmentative biological control and IPM strategies against these pests.

## Figures and Tables

**Table 1:** Mean (±SE) duration of the pre-imaginal developmental period and survival of *Chrysoperla externa* consuming different prey items: *Diatraea saccharalis* eggs, *Leucoptera coffeella* pupae, and *Ephestia kuehniella* eggs.

Prey Item	N ^a^	Egg Stage (Days) ^b^	Larval Stage (Days) ^b^	Pupal Stage (Days) ^b^	Pre-Imaginal Period (Days) ^b^	Pre-Imaginal Survival (%) ^c^
*D. saccharalis*	21	4.7 ± 0.1 ^a^	27.3 ± 0.1 ^a^	13.6 ± 0.2 ^a^	46.6 ± 1.0 ^a^	26.3 ^a^
*L. coffeella*	18	5.4 ± 0.1 ^a^	17.7 ± 1.2 ^b^	9.8 ± 0.3 ^c^	36.0 ± 0.7 ^b^	22.5 ^a^
*E. kuehniella*	30	5.2 ± 0.1 ^a^	14.3 ± 0.4 ^c^	12.1 ± 0.5 ^b^	32.1 ± 0.7 ^c^	37.5 ^a^

^a^ Number of individuals that completed the pre-imaginal period. ^b^ Means followed by the same letter within a column are not significantly different according to the DSCF test for multiple comparisons (*p* > 0.05). ^c^ Means followed by the same letter within a column are not significantly different according to logistic regression (*p* > 0.05).

**Table 2:** Mean adult longevity (±SE), female fecundity (±SE), and egg hatching rate of *Chrysoperla externa* consuming different prey items: *Diatraea saccharalis* eggs, *Leucoptera coffeella* pupae, and *Ephestia kuehniella* eggs.

Prey Item	N (♀/♂) ^a^	Female Longevity (Days) ^b^	Male Longevity (Days) ^b^	Fecundity (Eggs/Female) ^b^	Egg Hatching Rate (%) ^c^
*D. saccharalis*	19 (10/9)	49.3 ± 5.3 ^a^	30.3 ± 9.7 ^b^	199.4 ± 81.0 ^a^	65.0 ^a^
*L. coffeella*	18 (9/9)	58.0 ± 2.7 ^a^	74.5 ± 1.2 ^a^	205.3 ± 42.8 ^a^	76.0 ^a^
*E. kuehniella*	28 (12/16)	29.7 ± 3.1 ^b^	26.9 ± 4.2 ^b^	397.3 ± 70.3 ^a^	45.0 ^b^

^a^ Number of adults evaluated; ♀ = females and ♂ = males. ^b^ Means followed by the same letter within the column are not significantly different according to the Student–Newman–Keuls test (*p* > 0.05). ^c^ Egg hatching rate = (total number of eggs hatched/initial number of tested eggs) × 100. Means followed by the same letter within the column are not significantly different according to the logistic regression (*p* > 0.05).

**Table 3:** Mean pre-oviposition (±SE) and oviposition (±SE) periods of *Chrysoperla externa* consuming different prey items: *Diatraea saccharalis* eggs, *Leucoptera coffeella* pupae, and *Ephestia kuehniella* eggs.

Prey Item	N (♀) ^a^	Pre-Oviposition (Days) ^b^	Oviposition (Days) ^b^
*D. saccharalis*	10	5.5 ± 0.3 ^a^	31.7 ± 7.0 ^b^
*L. coffeella*	9	5.1 ± 0.4 ^a^	57.7 ± 12.1 ^a^
*E. kuehniella*	12	5.3 ± 0.2 ^a^	24.4 ± 3.6 ^b^

^a^ Number of adults evaluated, ♀ = females. ^b^ Means followed by the same letter within the column are not significantly different according to the Student–Newman–Keuls test (*p* > 0.05).

**Table 4:** Means (±SE) of demographic parameters of *Chrysoperla externa* consuming different prey items: *Diatraea saccharalis* eggs, *Leucoptera coffeella* pupae, and *Ephestia kuehniella* eggs.

Prey Item	N ^a^	*R*_0_ (♀/♀) ^b^	*r*_m_ (♀/♀/Day) ^b^	*λ* (♀/Day) ^b^	*T* (Days) ^b^	*Dt* (Days) ^b^
*D. saccharalis*	9	51.8 ± 8.3 ^b^	0.069 ± 0.003 ^b^	1.071 ± 0.007 ^b^	58.9 ± 3.1 ^a^	10.1 ± 0.3 ^a^
*L. coffeella*	9	46.2 ± 4.5 ^b^	0.074 ± 0.004 ^b^	1.076 ± 0.004 ^b^	51.4 ± 2.5 ^ab^	9.3 ± 0.4 ^a^
*E. kuehniella*	9	149.0 ± 21.3 ^a^	0.125 ± 0.006 ^a^	1.131 ± 0.009 ^a^	40.1 ± 1.3 ^b^	5.5 ± 0.2 ^b^

^a^ Number of females used initially. *R*_0_, net reproductive rate (female offspring per female). *r*_m_, intrinsic rate of population growth. *λ*, finite rate of population growth. *T*, average generation time. *Dt*, time for the population to double in number. ^b^ Means followed by the same letter within a column are not significantly different according to the Student’s *t* test for pairwise comparisons (*p* < 0.05). The Jackknife method was used to calculate standard errors.

## Data Availability

The data presented in this study are available on request from the corresponding author (A.M.V.).

## Supplementary Materials

The following supporting information can be downloaded at: https://www.mdpi.com/article/10.3390/insects15050330/s1, Figure S1: Prey items offered to *Chrysoperla externa* larvae for consumption: (a) *Diatraea saccharalis* eggs, (b) *Leucoptera coffeella* pupae, and (c) *Ephestia kuehniella* eggs.
