# Supplementary material for: Testing the Effects of Prey Type on the Life History and Population-Level Parameters of Chrysoperla externa (Neuroptera: Chrysopidae)
Source: Insects. 2024 May 3;15(5):330. doi: 10.3390/insects15050330 (PMC11122253; doi:10.3390/insects15050330)

Figure S1. Prey items offered to *Chrysoperla externa* larvae for consumption: (a) *Diatraea saccharalis* eggs, (b) *Leucoptera coffeella* pupae, and (c) *Ephestia kuehniella* eggs.

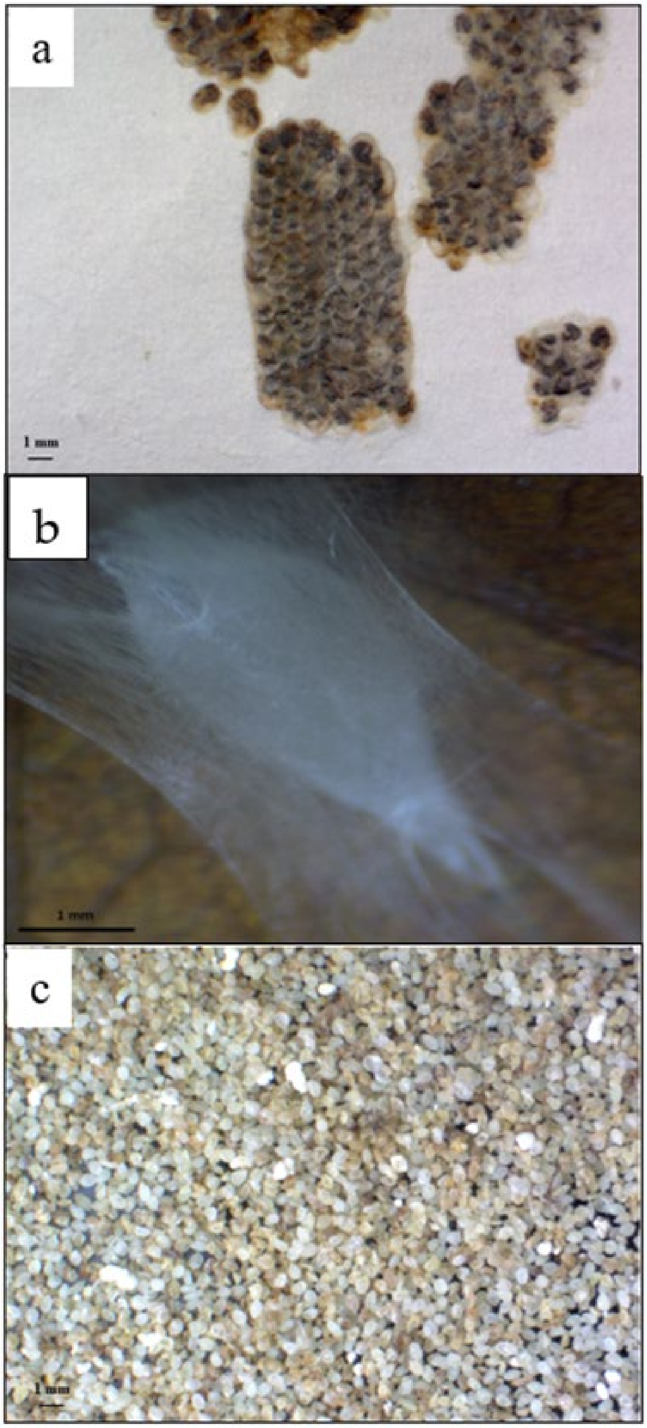

Supplement: Supplementary file 1 [file insects-15-00330-s001.zip › insects-2953323-supplementary.pdf]
